# Deficiency of Lipoprotein Lipase in Neurons Decreases AMPA Receptor Phosphorylation and Leads to Neurobehavioral Abnormalities in Mice

**DOI:** 10.1371/journal.pone.0135113

**Published:** 2015-08-11

**Authors:** Tian Yu, Matthew D. Taussig, Nicholas V. DiPatrizio, Giuseppe Astarita, Daniele Piomelli, Bryan C. Bergman, Mark L. Dell’Acqua, Robert H. Eckel, Hong Wang

**Affiliations:** 1 Division of Endocrinology, Metabolism, and Diabetes, Department of Medicine, University of Colorado, School of Medicine, Aurora, CO 80045, United States of America; 2 Department of Pharmacology, University of California Irvine, CA 92617, United States of America; 3 Department of Pharmacology, University of Colorado, School of Medicine, Aurora, CO 80045, United States of America; Monash University, AUSTRALIA

## Abstract

Alterations in lipid metabolism have been found in several neurodegenerative disorders, including Alzheimer’s disease. Lipoprotein lipase (LPL) hydrolyzes triacylglycerides in lipoproteins and regulates lipid metabolism in multiple organs and tissues, including the central nervous system (CNS). Though many brain regions express LPL, the functions of this lipase in the CNS remain largely unknown. We developed mice with neuron-specific LPL deficiency that became obese on chow by 16 wks in homozygous mutant mice (NEXLPL-/-) and 10 mo in heterozygous mice (NEXLPL+/-). In the present study, we show that 21 mo NEXLPL+/- mice display substantial cognitive function decline including poorer learning and memory, and increased anxiety with no difference in general motor activities and exploratory behavior. These neurobehavioral abnormalities are associated with a reduction in the 2-amino-3-(3-hydroxy-5-methyl-isoxazol-4-yl) propanoic acid (AMPA) receptor subunit GluA1 and its phosphorylation, without any alterations in amyloid β accumulation. Importantly, a marked deficit in omega-3 and omega-6 polyunsaturated fatty acids (PUFA) in the hippocampus precedes the development of the neurobehavioral phenotype of NEXLPL+/- mice. And, a diet supplemented with n-3 PUFA can improve the learning and memory of NEXLPL+/- mice at both 10 mo and 21 mo of age. We interpret these findings to indicate that LPL regulates the availability of PUFA in the CNS and, this in turn, impacts the strength of synaptic plasticity in the brain of aging mice through the modification of AMPA receptor and its phosphorylation.

## Introduction

Neurodegenerative diseases such as Alzheimer’s disease (AD) that are characterized by a progressive cognitive deficit, are quickly becoming an alarming health problem all over the world as the general population life span continues to increase in the past several decades. Although a plethora of studies have addressed the causes and treatment of these diseases, minimal progress has ensued. Alterations in brain lipids including phospholipids and glycolipids have been demonstrated in neurodegenerative disorders such as AD [1, 2]. The altered lipid metabolism in AD may relate to the changes in central nervous system (CNS) lipoprotein metabolism, i.e. increases in apolipoproteins J [3] and D [4], and the VLDL receptor [5], all found in the senile plaque. The excessive accumulation of amyloid β (Aβ) in senile plaques in the brain is a widely accepted hallmark of AD. Apolipoprotein E (ApoE) is a known amyloid β binding protein, and ApoE4, an allele of the apoE gene, is a major genetic risk factor for AD [6]. Furthermore, lipoprotein receptors such as the low-density lipoprotein receptor-related protein 1 (LRP1) have also been implicated to play a role in Aβ processing and clearance [7]. Increasing evidence has also suggested a role for lipoproteins and their receptors in the regulation of body weight and energy balance in addition to neurobehavioral functions [8, 9].

Lipoprotein lipase (LPL) binds ApoE and LRP1, and has also been localized in senile plaques [10]. LPL is a multifunctional enzyme produced by many tissues and is rate limiting for the cellular uptake of fatty acids following the hydrolysis of circulating triacylglyceride (TG)-rich lipoproteins for lipid storage and/or oxidation in peripheral tissues [8]. LPL is present throughout the CNS, but little is known about its physiological function and the importance of TG-rich lipoprotein metabolism in the brain. Whole body LPL knockout mice rescued by somatic gene transfer have been used as a model to study the importance of LPL in cognitive function. These mice displayed learning and memory deficits which were associated with a defect in synapse vesicle recycling [11]. However, this model is problematic in that the systemic metabolic disturbances that occur when peripheral LPL is deficient remain, and the observed phenotype may be in part independent of neuronal LPL deficiency. More recently, LPL has been reported as an Aβ binding protein that could facilitate Aβ clearing by astrocytes *in vitro*[12]. However, the physiological relevance of this finding remains unclear due to the lack of appropriate animal models.

We recently created neuron-specific LPL deficient mice (NEXLPL-/-) that develop obesity by 16 wks on standard chow, and display marked reductions in omega-3 polyunsaturated fatty acids in the hypothalamus at 3 months, before the onset of obesity [13]. Heterozygous neuron-specific LPL deficient mice (NEXLPL+/-) also develop obesity on a delayed time course, and herein we use aged NEXLPL+/- mice to show for the first time that mice with neuron-specific deficiency of LPL display learning and memory deficits in addition to other behavioral abnormalities, clearly demonstrating the importance of neuronal LPL in cognitive function.

## Materials and Methods

### Animal Model, Measurement of Body Weight, Body Composition and Plasma Metabolic Parameter

All animal protocols have been approved by University of Colorado Denver/Anschutz Medical Campus Institutional Animal Care and Use Committee (protocol number: 97813(05)1D). The generation of the mice has been described [13]. Body composition was measured on anesthetized mice by dual-energy X-ray absorptiometry using a mouse densitometer (PIXImus2, Lunar Corp.). Plasma samples were collected after a 4 hr fast, and metabolic parameters were measured using Milliplex map Kit (Millipore) and as previously described [14]. Only female mice were used in this study due to the limited number of male mice available for study at older ages. The age of mice was between 21–24 mo unless specified otherwise. Littermate controls (NEXCreLPL+/+, CTR) were used in studies at all ages.

### Quantitative Real-Time PCR

Hippocampus was collected into RNAlater (Qiagen) from 21~24 month old anesthetized mice after a 4 hr fast and stored at 4°C. Total RNA was extracted and reverse transcribed as previously described [13]. Quantitative PCR was performed using primer sets for genes of interest, two reference genes and iQ Supermix or iQ SYBR Supermix (Bio-Rad) following the manufacturer’s protocols.

### Behavioral tests

For Open Field test, total distance traveled and average speed in the open field chamber (28 x 28 cm) was quantified during 15 min under standard room lighting conditions. Novel Object Recognition apparatus was the same dimension as in Open Field test. The mice were habituated in the apparatus the day before the test day. On the test day, mice were put in the apparatus 5 min per trial, and 2 trials per animal were performed with an hour break between each trial. The objects were painted with different colors and were presented in shuffled sequence to minimize the effect of preferences of shape/color. Recorded videos were analyzed by ANY-maze (Stoelting, USA) to distance, speed and time around subjects. The discrimination ratio = (Tnew–Told)/(Tnew+Told).

Morris Water Maze was performed in a circular tank (120 cm diameter, 45 cm tall) with a circular escape platform (10 cm in diameter) located either 1 cm below (invisible platform) or above (visible platform) the water surface. The latency of finding the platform was recorded for each trial. On day 1, mice were acclimated to the maze during a 4-trial habituation session with the platform visible. From day 4 to 8, mice were tested for 4 trials per day in similar fashion but with the platform invisible. On day 11, after a 2 days break, the mice were tested for their memory of the platform position. On day 13, the mice were tested with the platform in a different location.

For the Morris Water Maze after diet feeding, 3–5 mo or 18–20 mo old NXLPL+/- mice were fed with a high carbohydrate diet (HC diet, 70% carbohydrate by kcal, 10% fat by kcal, omega-3 to omega-6 ratio 1:7; D12450B from Research Diets) or high carbohydrate diet enriched in omega-3 fatty acids (105.1 g polyunsaturated fatty acids per kg HC diet, omega-3 to omega-6 ratio 3:1, other macronutrient matched to D12450B, custom made from Research Diets) for 7 or 3 mo before the water maze test was done at 10–12 mo or 21–23 mo of age, specifically.

To measure the swimming speed of CTR (on chow diet), CTR (on high fat diet, HF diet, 45% fat by kcal, D12451, Research Diet) and NEXLPL+/- (on HF diet), the mice were put into the pool without platform and were video recorded and further analyzed by TopScan (CleverSys, USA).

The apparatus used for the Elevated Plus Maze test was in the configuration of a ‘+’ shape and comprises two open arms (30 x 5 x 0.5 cm) across from each other and perpendicular to two closed arms (30 x 5 x 15 cm) with a center platform (5 x 5 x 0.5 cm). Mice were put in the maze for 10 min. TopScan (CleverSys, USA) was used to track the movement of mice, and time spent in each arm was recorded for analysis.

### Measurement of amyloid β

For the determination of insoluble Amyloid β levels by ELISA, mice half brains were homogenized in ice-cold PBS containing 5 M guanidine / 50 mM Tris HCl (pH8.0) and protease inhibitor. Homogenates were mixed for 3–4 h at room temperature and centrifuged at 16,000 g for 20 min at 4°C. For soluble Aβ, mice half brains were homogenized in 250 mM sucrose, 20 mM Tris-HCl (pH7.4), 1mM EDTA, 1 mM EGTA supplemented with protease inhibitor at 4°C. Same volume of 0.4% of diethylamine and tissue sucrose homogenate was mixed, centrifuged at 100,000g for 1 h at 4°C. Both supernatants were tested with an ELISA kit for mouse Aβ42 (Invitrogen).

### Western Blot

Mice hippocampi and cerebellums were collected and lysed with ice cold lysis buffer supplemented with 1% protease inhibitor cocktail (Pierce) and phosphatase inhibitor (100 uM NaF and 0.5 mM active Sodium orthovanadate). Protein concentrations were determined by Pierce BCA protein assay kit (Thermo Scientific), and the same amount of total protein was loaded to each lane. The quantification of target protein in each lane was normalized to the total amount of protein, which is determined by Ponceau S staining. Antibodies used included GluA1, p-GluA1, GluN1, PSD95 (all from Cell Signaling), and synaptophysin (Millipore).

### LC/MS and GC/MS Lipidomic Analyses of Brain Tissues

The detailed method of lipidomic analyses has described before [13, 15]. Briefly, mouse brains were quick-frozen in 2-methylbutane at -40°C and then stored at -80°C until further processing. Brain hippocampus was punched from frozen brains, which were weighed and homogenized in methanol methanol:chloroform (1:2) containing the following internal standards: d_8_-arachidonic acid, d_8_-2-arachidonoyl glycerol (Cayman Chemical), diheptadecanoin, trinonadecenoin, triheptadecanoin (Nu-Chek Prep) and extracted with water. For LCMS, organic phases were collected and dried under liquid nitrogen. Lipids were reconstituted in chloroform/methanol (1:4) for liquid chromatography/mass spectrometry (LCMS) analyses use an Agilent 1100-LC system (Agilent Technologies, Palo Alto, CA) [16]. For GCMS, dried organic phases were re-suspended in chloroform and added to aminopropyl solid-phase extraction columns (Discovery DSC-NH2, Supelco). FFAs were isolated using solid-phase extraction based on the methods originally described by Kaluzny et al. [17], converted to fatty acid methyl esters (FAME), and stable isotope ratios of ^17^C in FAMEs were measured using gas chromatography/mass spectrometry (GCMS) system (Thermo Electron, Bremen, Germany). Weighted samples of Chow diet (Teklad rodent diet 2018, 18% fat content, Harlan Laboratories, Denver, CO) and HC diet (Research Diets D12450B, details stated above) diets underwent lipid extraction and the samples were submitted for GC/MS lipidomic analysis as described above for brain samples.

### Statistical Analyses

Results are presented as mean ± SEM. Wilcoxon signed-rank test was used for novel object recognition test. One way repeated measure ANOVA was performed for Morris Water Maze data. Student t-tests were performed for all the other statistical analysis. P values less than 0.05 were considered significant.

## Results

### Metabolic characterization of 21–24 mo NEXLPL+/- mice

**Fig 1:**
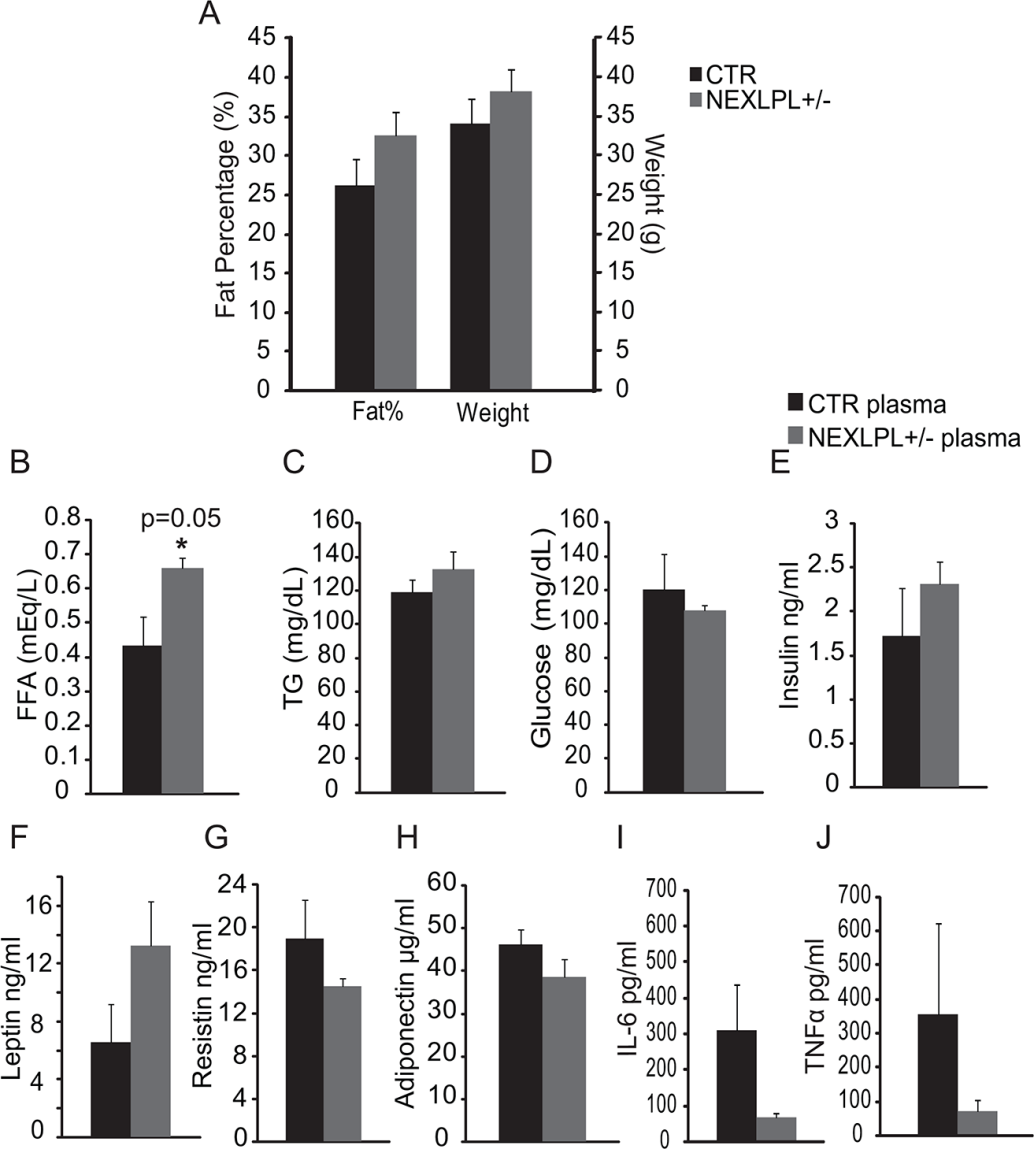
Metabolic profile of 21–24 mo NEXLPL+/- mice. (A) Body composition and weight (CTR n = 8; NEXLPL+/- n = 7) (B)-(E) Plasma levels of free fatty acid (FFA), triglyceride (TG), glucose and insulin (CTR n = 5; NEXLPL+/- n = 4) (F)-(J) Plasma levels of leptin, resistin, adiponectin, IL-6 and TNFα (CTR n = 5; NEXLPL+/- n = 4).

We have previously shown that NEXLPL-/- mice were hyperphagic and became obese by 16 wks, and displayed reductions in omega-3 polyunsaturated fatty acids in the hypothalamus before the onset of obesity [13]. In the present study, we evaluated the metabolic profile of 21–24 mo old NEXLPL+/- mice. Younger NEXLPL+/- mice were reported to follow a similar obesity development path as their homozygous counterparts, only with a later onset. By 12 months of age, NEXLPL+/- mice were obese with more fat mass, similar to NEXLPL-/- mice at 6 mo [13]. However, at 21+ mo, the increases in body weight and fat mass percentage became non-significant, but still trending higher in NEXLPL+/- mice (Fig 1A). This is most likely due to the aging of mice in general and the random spontaneous development of obesity often seen in older control (CTR) mice. Noticeably, older NEXLPL+/- mice displayed higher plasma free fatty acid (FFA) concentrations (Fig 1B, p = 0.05) but normal fasting plasma TG (Fig 1C), glucose (Fig 1D) and insulin levels (Fig 1E). The increase in FFA was consistent with a previously reported trend in 6 mo NEXLPL+/- mice [13]. Fasting plasma leptin was also trending higher (Fig 1F), while resistin (Fig 1G) and adiponectin (Fig 1H) levels were not significantly different from the controls. Interestingly, the two inflammatory cytokines IL-6 (Fig 1I) and TNFα (Fig 1J), usually elevated in the obese state, were trending lower in NEXLPL+/- mice compared to controls.

### NEXLPL+/- mice exhibit multiple neurobehavioral abnormalities

**Fig 2:**
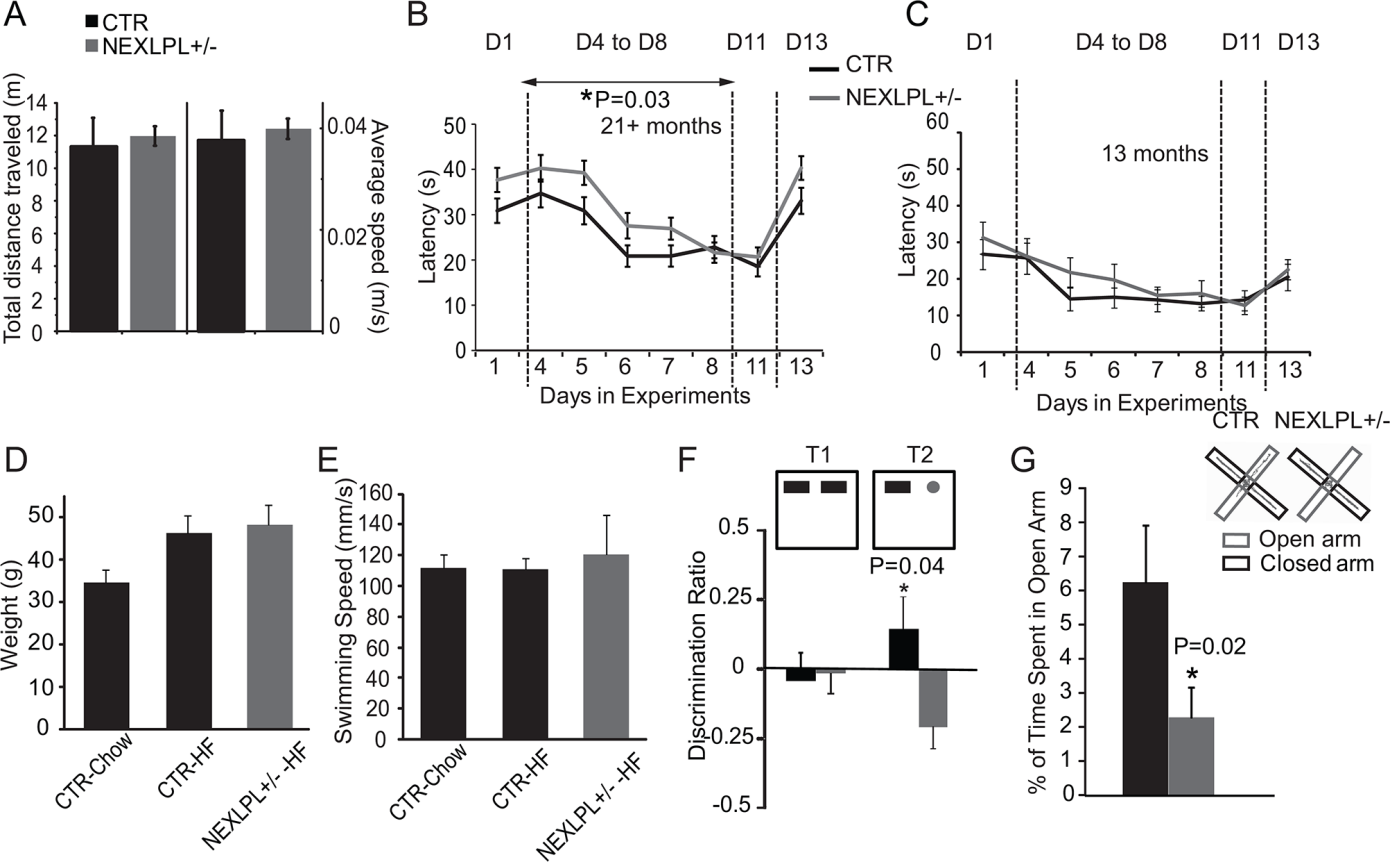
NEXLPL+/- mice have multiple neurobehavioral abnormalities. (A) Motor activities were tested in an open field apparatus (CTR n = 9; NEXLPL+/- n = 12). (B) Spatial learning and memory was tested with Morris water maze at 21+ mo age: Day 1: visible platform; Day 4–8: hidden platform, learning phase; Day 11: hidden platform, short memory; Day13: platform position changed. From Day 4 to Day 8, one way repeated measure ANOVA showed genotype difference, p<0.01, (CTR n = 14; NEXLPL+/- n = 15). (C) Spatial learning and memory was tested with Morris Water Maze described above at 13 mo age (CTR n = 11; NEXLPL+/- n = 12). (D-E) Measurement of weights and swimming speeds of chow-fed CTR (n = 4), high fat (HF)-fed CTR (n = 6), and HF-fed NEXLPL+/- (n = 4) mice. (F) Recognition memory was tested with novel object recognition. The test is performed as 2 trials. In trial 1 (T1), two identical objects were placed in the apparatus, followed by trial 2 (T2) with replacement of one object. Discrimination ratio = (Tnew-Told)/(Tnew+Told) (CTR n = 9; NEXLPL+/- n = 10, p = 0.04). (G) Elevated plus maze: Inset is the representative tracts of mice. The apparatus was 50 cm above the ground with two open arms and two closed arms (CTR n = 9; NEXLPL+/- n = 11, p = 0.02).

To investigate potential neurobehavioral abnormalities in NEXLPL+/- mice, we challenged 21–24 mo old NEXLPL+/- and CTR mice with a battery of tests that evaluate learning, memory and anxiety. The general exploratory behavior and motor function of NEXLPL+/- mice was first investigated in an open field test. Both CTR and NEXLPL+/- mice moved at similar speeds and traveled a similar distance during the time period of the test (Fig 2A).

The Morris Water Maze test, a classical spatial learning and memory test that is mostly linked to hippocampal function, was conducted on 21–24 mo NEXLPL+/- and CTR mice. Importantly, Nissl stain of the hippocampus showed no obvious developmental and/or structural abnormalities in NEXLPL+/- mice (data not shown). NEXLPL+/- mice displayed similar ability to find the visible platform on Day 1 of the experiment (Fig 2B, D1), but exhibited significantly increased latency to find the hidden platform during the entire 5-day hidden platform period (Fig 2B, D4-D8, p = 0.03). However, by the end of the learning period, NEXLPL+/- mice found the hidden platform as quickly as CTR mice (Fig 2B, D8). After a 2-day break we further tested long-term memory on day 11 (Fig 2B, D11), and the ability to find a new platform position on day 13 (Fig 2B, D13). NEXLPL+/- mice displayed no difference in long term memory but a trend of latency in adaptation to find the new platform. Together, these results are suggestive of a deficit in spatial learning and memory in NEXLPL+/- mice. The deficit was not observed in younger NEXLPL+/- mice (6 mo: data not shown and 13 mo: Fig 2C).

Even though the body weight of NEXLPL+/- mice was not significantly higher than CTR mice, we still conducted a control experiment to rule out the potential impact of trending higher body weight and fat mass on swimming speed. A group of 15 mo CTR and NEXLPL+/- mice were fed a high-fat (HF) diet for 3 mo to induce weight gain in CTR mice that was similar to the average weight difference between chow-fed NEXLPL+/- and CTR mice (Fig 2D). A simple swimming speed test was conducted in the Morris Water Maze pool for 1 min. Despite the heavier body weights of HF-fed CTR and NEXLPL+/- mice (compared to chow-fed CTR), the swimming speeds were similar among all three groups (Fig 2E). Thus the difference in latency observed in Morris Water Maze above cannot be attributed to differences in body weight.

Novel object recognition is a behavioral test that is widely used to test recognition memory, a complex process that involves several brain regions, including the perirhinal cortex, medial prefrontal cortex and hippocampus [18]. We utilized the simplest version of this test to discriminate between the hippocampus-independent, cortex-based recognition memories in NEXLPL+/- and CTR mice. As shown in Fig 2F, when we introduced novel objects to the apparatus at the same position, CTR mice always spent more time around the novel objects, relative to NEXLPL+/- mice. This result indicates impairment in recognition memory function in NEXLPL+/- mice.

To identify other potential behavioral abnormalities in NEXLPL+/- mice, we turned to the elevated plus maze (EPM) to test anxiety of the animals. The EPM tests the innate fear of mice for predatory birds and involves multiple regions of the limbic system (hippocampus, amygdala) as well as the dorsal raphe nucleus [19]. When CTR and NEXLPL+/- mice were challenged on an EPM 50 cm above ground level, CTR mice spent more time in the closed arm, but did explore the open arm from time to time (Fig 2G). NEXLPL+/- mice spent much less time in the open arm than did CTR animals (Fig 2G, 6.2% for CTR vs. 2.3% for NEXLPL+/- time spent in Open Arm, p = 0.02), indicating an increased level of anxiety.

In summary, three different behavioral tests indicate that neuronal LPL deficiency in the CNS results in multiple behavioral abnormalities in aging mice. Moreover, at least two behavioral tests suggest that the cognitive deficit in mice lacking neuronal LPL might be underpinned by impairments in hippocampal function.

### Amyloid-β clearing and common lipid pathway gene expression are normal in NEXLPL+/- mice

**Fig 3:**
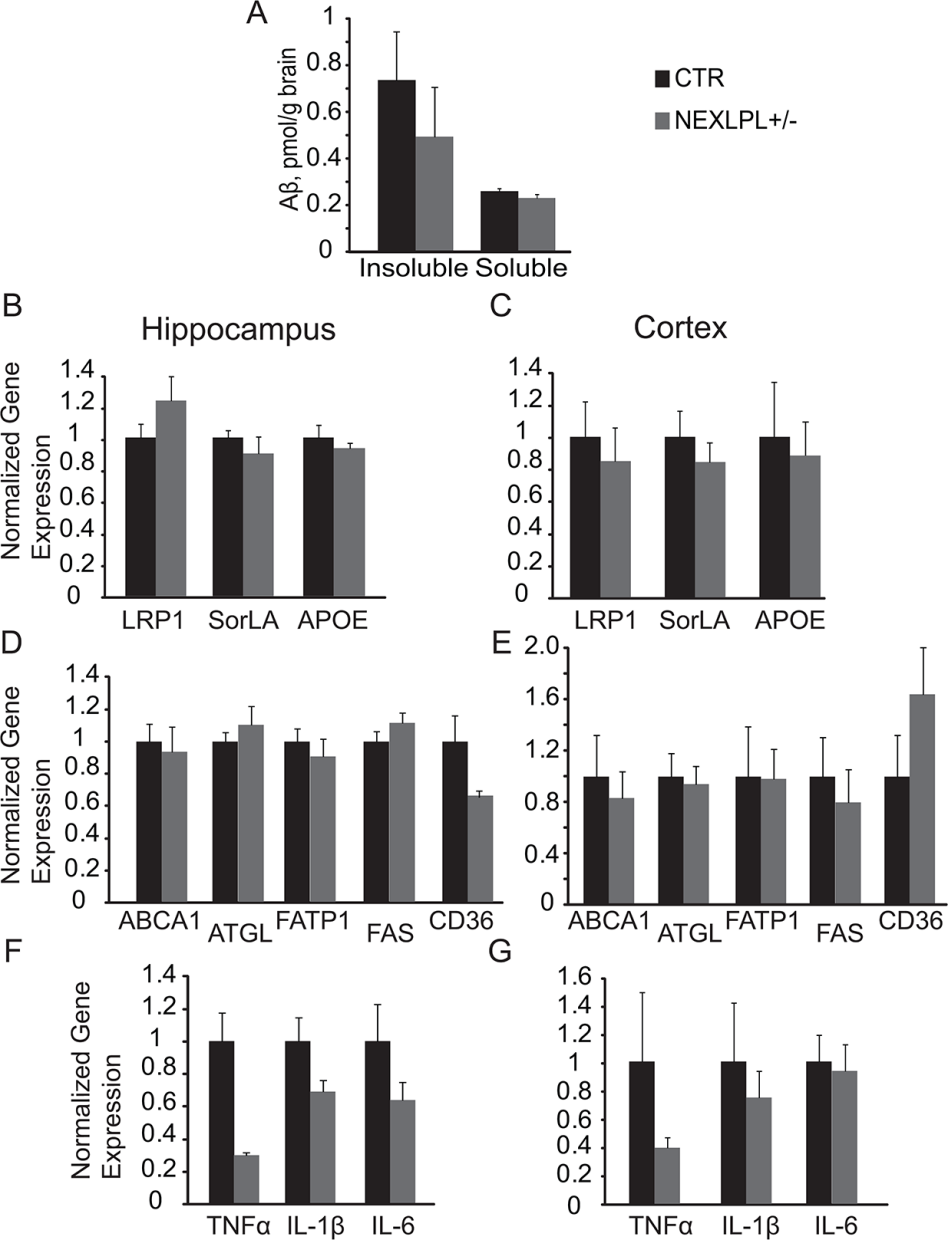
Gene expression of NEXLPL+/- mice hippocampus and cortex. (A) Whole brain Amyloid β peptide in 21–24 mo NEXLPL+/- mice (CTR n = 4; NEXLPL+/- n = 4). (B)(D)(F) Gene expression in the hippocampus of NEXLPL+/- mice at 21–24 mo (CTR n = 5; NEXLPL+/- n = 5). (C)(E)(G) Gene expression in the cortex of NEXLPL+/- mice at 21–24 mo (CTR n = 5; NEXLPL+/- n = 5).

AD is notoriously accompanied by the deposition of neurotoxic amyloid-β (Aβ) peptides. Since polymorphisms in LPL have been shown to relate to the presence of neurofibrillary tangles and senile plaque densities in the brains of patients with AD [20], we examined whether NEXLPL+/- mice might have an excessive accumulation of Aβ in the brain. As shown in Fig 3A, the levels of both insoluble and soluble Aβ were normal in the brain of NEXLPL+/- mice.

Next we used real-time PCR to evaluate the mRNA levels of various known genes in lipid pathways that have been implicated in regulating brain function. In both the hippocampus and cortex, the highly AD associated genes LRP1, SorLA (a sorting lipoprotein receptor) and ApoE were comparable in NEXLPL+/- and CTR mice (Fig 3B and 3C). A selected group of lipid processing/binding protein genes including ATP-binding cassette transporter 1 (ABCA1), adipose triglyceride lipase (ATGL), fatty acid transport protein 1(FATP1), fatty acid synthase (FAS) and CD36 were examined in hippocampus (Fig 3D) and cortex (Fig 3E). Although levels of CD36 mRNA trended lower in the hippocampus, expression of all other lipid-processing genes in both hippocampus and cortex was similar between NEXLPL+/- and CTR mice. Interestingly, gene expression of the inflammatory cytokines TNFα, IL-1β and IL-6 trended lower in the hippocampus (Fig 3F) of NEXLPL+/- mice, whereas in cortex, only TNFα and IL-1β trended lower (Fig 3G).

Together, these gene expression results along with the trending lower levels of TNFα in fasting plasma (Fig 1J) do not support a role of either Aβ clearing or increased inflammation in the hippocampus as the mechanism for cognitive impairment in NEXLPL+/- mice.

### Reduction of AMPA receptor subunit GluA1 and its phosphorylation in hippocampus as a potential mechanism for cognitive impairment in NEXLPL+/- mice

The most LPL deficient brain region in NEXLPL-/- mice is the hippocampus [8, 13], which is implicated in more than one test underlying the behavioral abnormalities in NEXLPL+/- mice. Because alterations in synaptic function may be the mechanism behind the learning deficits in somatic gene rescued LPL deficient mice [11], we investigated the proteins involved in synaptic plasticity in NEXLPL+/- mice.

**Fig 4:**
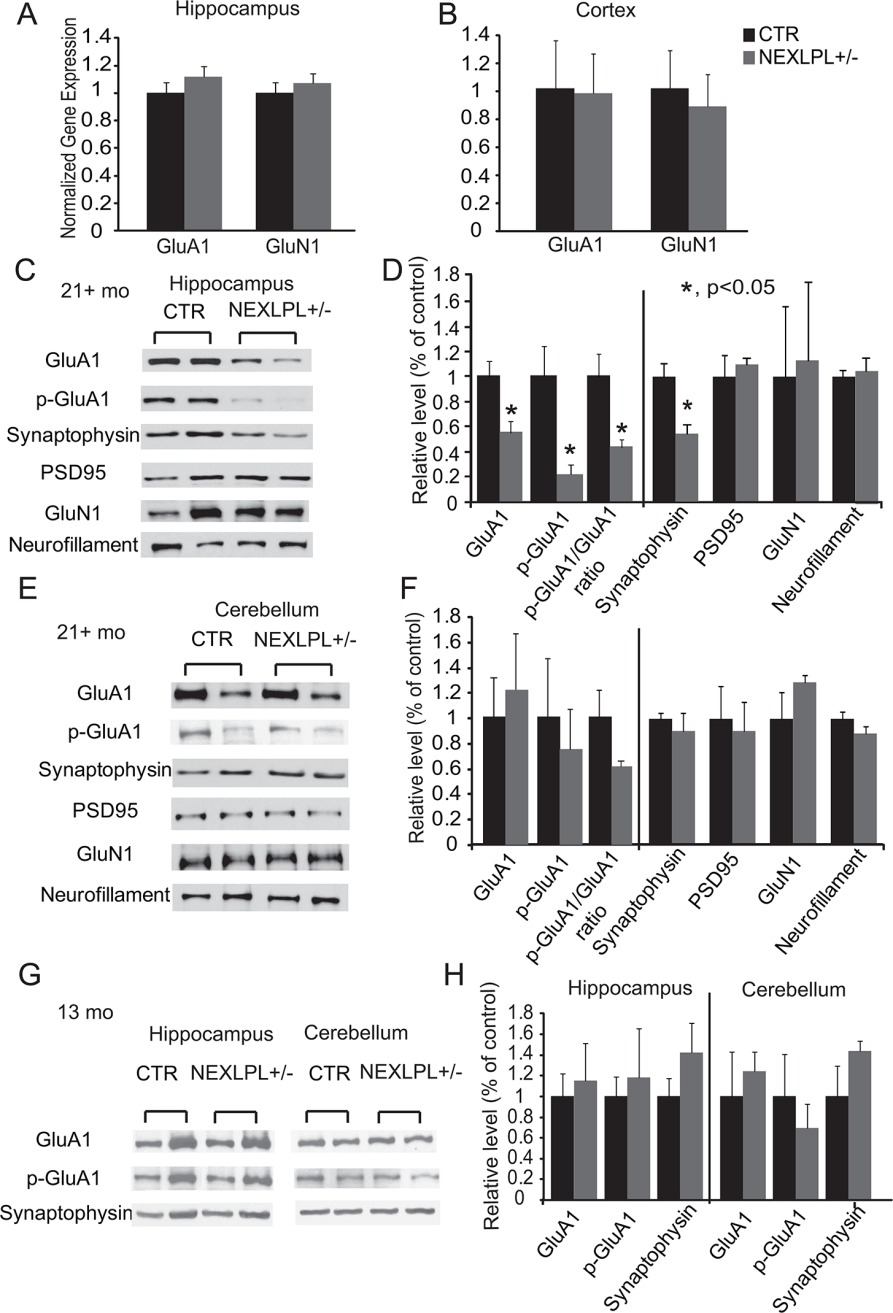
Gene and protein expression of synaptic markers in brain regions of 21–24 mo NEXLPL+/- mice. (A) GluA1 and GluN1 gene expression in hippocampus (CTR n = 5; NEXLPL+/- n = 5). (B) GluA1 and GluN1 gene expression in cortex (CTR n = 5; NEXLPL+/- n = 5). (C)(D) Levels of GluA1 protein (CTR n = 6; NEXLPL+/- n = 7), phosphorylated GluA1 protein (CTR n = 6; NEXLPL+/- n = 7), synaptophysin (CTR n = 7; NEXLPL+/- n = 7), PSD95 (CTR n = 4; NEXLPL+/- n = 3), GluN1 (CTR n = 4; NEXLPL+/- n = 3) and neurofilament protein (CTR n = 4; NEXLPL+/- n = 3) in hippocampus of 21+ mo NEXLPL+/- mice by western blotting. (CTR n = 7; NEXLPL+/- n = 7) *, p<0.05. (E)(F) Levels of GluA1, p-GluA1, synaptophysin, PSD95, GluN1 and neurofilament protein in the cerebellum of 21+ mo NEXLPL+/- mice by western blotting (CTR n = 4; NEXLPL+/- n = 3). (G)(H) Levels of GluA1, p-GluA1 and synaptophysin protein in hippocampus and cerebellum of 13 mo NEXLPL+/- mice by western blotting (CTR n = 3; NEXLPL+/- n = 3).

The 2-amino-3-(3-hydroxy-5-methyl-isoxazol-4-yl) propanoic acid (AMPA) and N-methyl-D-aspartic acid (NMDA) receptors play essential roles in long-term potentiation (LTP)/long- term depression (LTD), which are widely considered as major cellular mechanisms underlying learning and memory [21]. The number of postsynaptic AMPA and NMDA receptors are important in defining synaptic strength [22, 23]. We found that transcription of genes encoding for the subunits of AMPAR (GluA1) and NMDAR (GluN1) was not different in hippocampus (Fig 4A) and cortex (Fig 4B) of NEXLPL+/- mice compared to CTR. However, when we examined protein levels of these receptors, a significant decrease in total GluA1 protein but not GluN1 protein was seen in the hippocampus of NEXLPL+/- mice (Fig 4C and 4D, p<0.05). In addition, a reduction in the pre-synaptic marker synaptophysin was also observed in NEXLPL+/- mice (Fig 4C and 4D, p<0.05), while the post-synaptic marker PSD95 and neurofilament protein levels were unchanged in NEXLPL+/- mice. Because specific phosphorylation of the AMPA receptor GluA1 at subunit Ser 845 is important for synaptic plasticity by preventing receptor degradation and stabilizing receptor membrane expression [24–29], we went on to show that GluA1 Ser 845 phosphorylation was decreased in NEXLPL+/- mice (Fig 4C and 4D, p = 0.005). The reduction in total GluA1, GluA1 phosphorylation and synaptophysin was specific to the hippocampus in aged NEXLPL+/- mice, with no such differences observed in protein extracts from the cerebellum of aged mice (Fig 4E and 4F) or younger NEXLPL+/- mice (13 mo) (Fig 4G and 4H).

### PUFA deficiency underlies the development of learning and memory deficit in NEXLPL+/- mice

**Fig 5:**
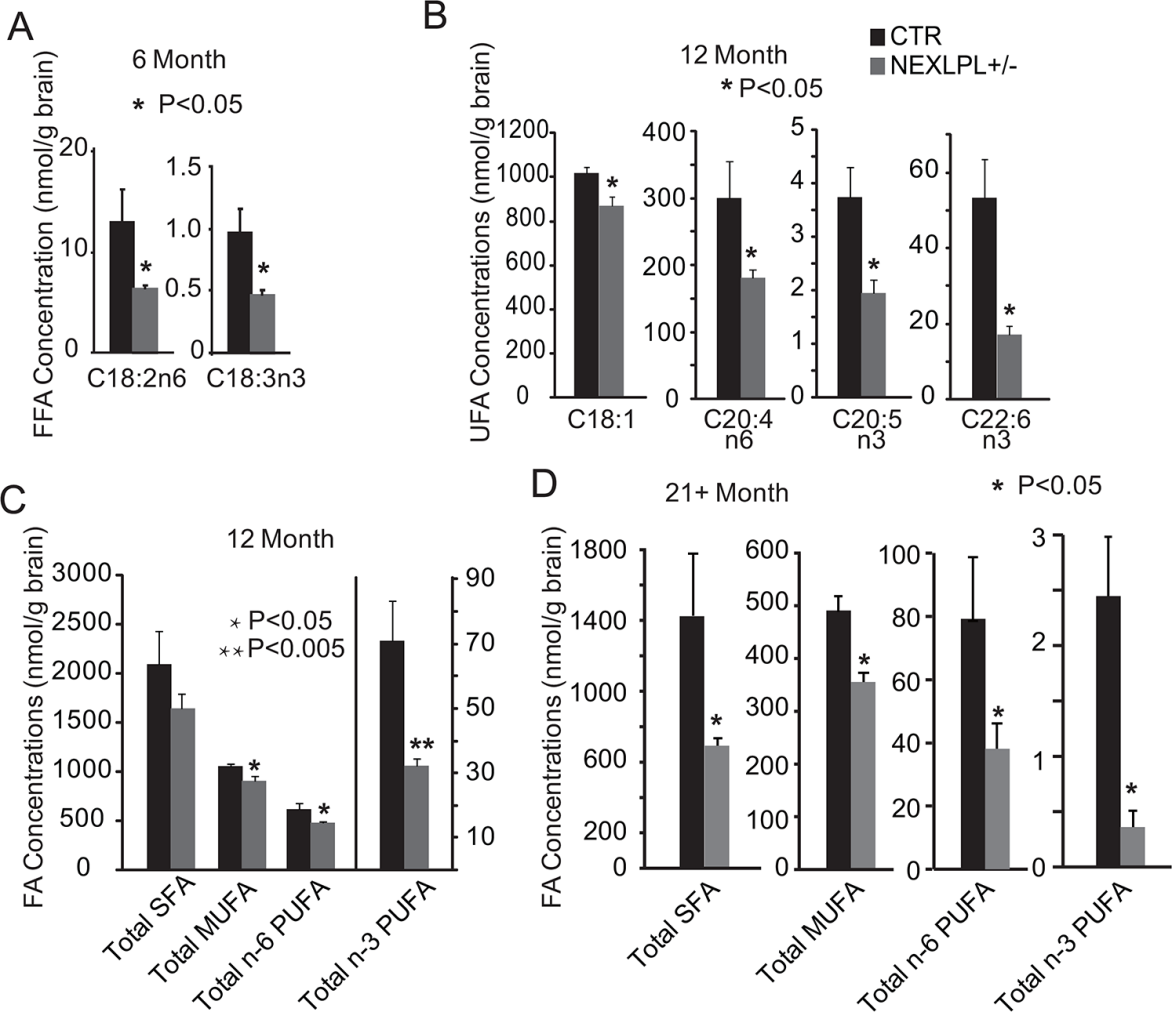
Hippocampal lipid metabolism in NEXLPL+/- and CTR mice at 6, 12 and 21+ months. (A) Reductions of C18:2n-3 and C18:3n-6 polyunsaturated fatty acids in the hippocampus of NEXLPL+/- mice at 6 mo (CTR n = 4; NEXLPL+/- n = 4, *, p<0.05). (B) Reductions of C18:1, C20:4–6, C20:5n-3, and C22:6n-3 fatty acids in the hippocampus of NEXLPL+/- mice at 12 mo (CTR n = 3; NEXLPL+/- n = 6, *, p<0.05). (C) Total saturated fatty acids (SFA), monounsaturated fatty acids (MUFA), n-6 and n-3 polyunsaturated fatty acids (PUFA) deficiencies of 12 mo NEXLPL+/- mice (CTR n = 3; NEXLPL+/- n = 6, *, p<0.05; **, p<0.005). (D) Total saturated fatty acids (SFA), monounsaturated fatty acids (MUFA), n-6 and n-3 polyunsaturated fatty acids (PUFA) deficiencies of 21+ mo NEXLPL+/- mice (CTR n = 3; NEXLPL+/- n = 5, *, p<0.05).

We have previously observed omega-3 PUFA deficiency in the hypothalamus of NEXLPL-/- mice at various ages, and proposed that this deficiency was linked to the development of obesity in NEXLPL-/- mice [13]. Targeted lipidomic analyses (Table 1) indicated that only two species of PUFA (18:2n-6 and 18:3n-3) were deficient at 6 mo in the hippocampus of NEXLPL+/- mice (Fig 5A, p = 0.01 and p = 0.03, respectively), while multiple species of more long chain omega-3 (20:5n-3 and 22:6n-3) and omega-6 PUFA (20:4n-6) were markedly deficient in the hippocampus of 12 mo NEXLPL+/- (Fig 5B, p = 0.01, p = 0.002 and p = 0.01, respectively), with the most significant deficiency in omega-3 PUFAs (Fig 5B and 5C, p<0.005). The omega-3 PUFA deficiency was further developed in 21+ mo NEXLPL+/- mice as shown in Fig 5D, along with a general deficiency in all types of FFAs in the hippocampus of 21 mo NEXLPL+/- compared to CTRs.

**Table 1:** Different species of FFA concentrations (nmol/g) of 6 mo and 12 mo NEXLPL+/- mice in hippocampus.

Hippocampus FFA		6 mo CTR		6 mo NEXLPL+/-			12 mo CTR		12 mo NEXLPL+/-		
Species	m/z	Average	SE	Average	SE	P-value	Average	SE	Average	SE	P-value
**C16:0**	255	38.7	6.2	37.3	2.4		1066.5	153.6	878.0	61.4	
**C18:0**	283	76.4	6.9	80.7	3.0		978.1	178.0	717.9	92.3	
**C20:0**	311	1.4	0.3	1.1	0.1		47.1	7.7	44.7	7.1	
**C16:1**	253	4.3	1.5	2.3	0.1		43.4	0.9	43.9	2.0	
**C18:1**	281	34.1	5.9	28.7	1.6		1018.9	24.9	870.5	40.0	0.045
**C18:2 n6**	279	12.9	1.9	6.25	0.2	0.01	316.6	8.0	301.5	4.7	
**C20:3 n6**	305	0.8	0.1	0.8	0.1		1.7	0.41	1.0	0.1	
**C20:4 n6**	303	45.2	3.1	51.3	4.0		300.6	55.10	180.7	12.9	0.01
**C18:3 n3**	277	1.0	0.2	0.45	0.0	0.03	13.2	0.03	12.7	0.3	
**C20:5 n3**	301	0.3	0.0	0.2	0.0		3.7	0.56	1.95	0.3	0.01
**C22:6 n3**	327	9.0	0.8	9.0	0.7		53.3	10.3	17.1	2.3	0.002

m/z: molecular weight of FFA. 6 mo: CTR n = 4, NEXLPL+/- n = 6; 12 mo: CTR n = 3, NEXLPL+/- n = 6.

**Fig 6:**
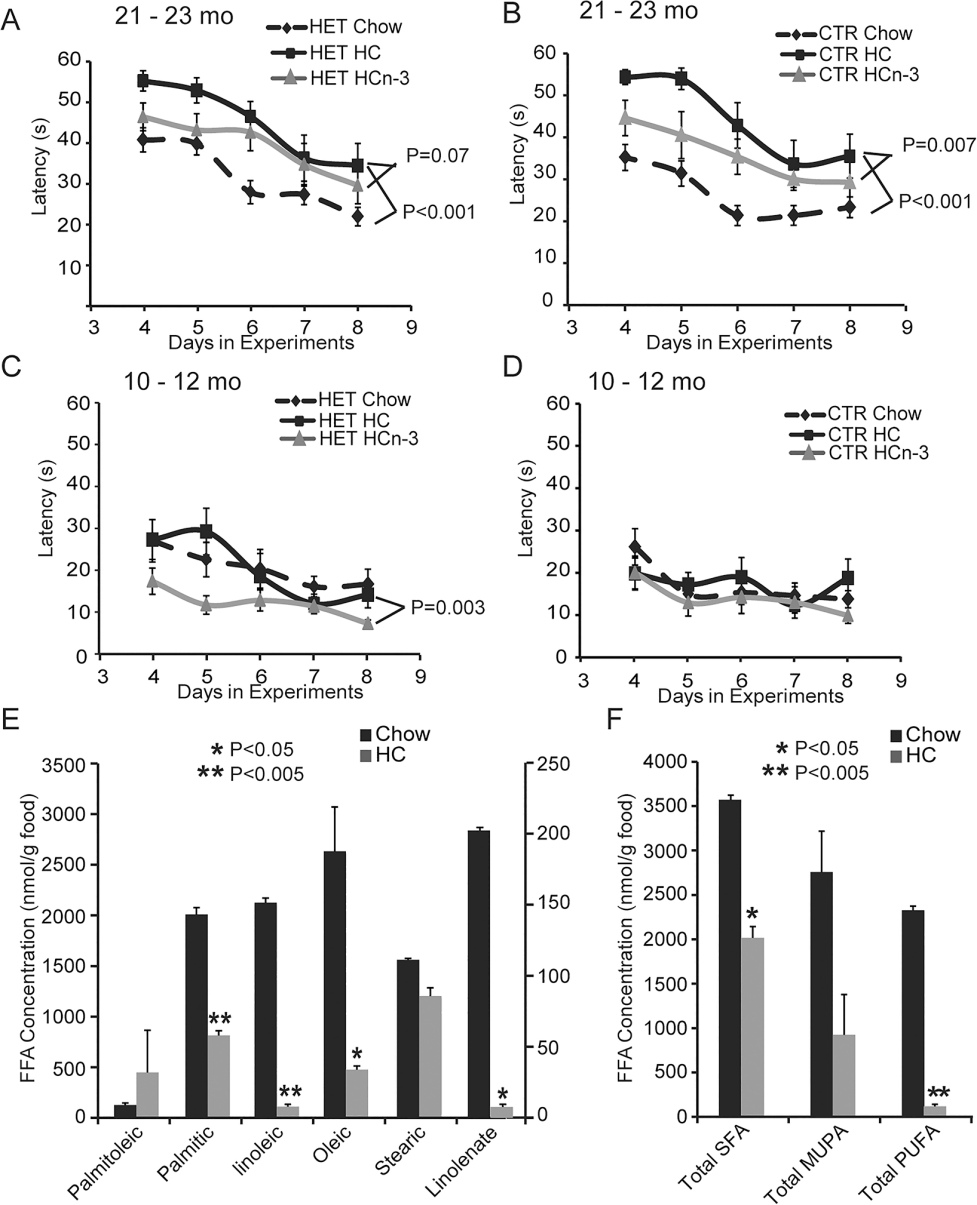
(A)(B) Spatial learning and memory was tested with Morris Water Maze of NEXLPL+/- and CTR mice at 21–23 mo of age. Mice were fed with HC or omega-3 fatty acid enriched HC diet for 3 mo from 19 mo of age. Day4-8: hidden platform, learning phase. One way repetitive measure ANOVA showed diet difference, HET on chow vs. HET on HC, p<0.001, CTR on chow vs. CTR on HC, p<0.001; HET on HCn-3 vs. HET on HC, p = 0.07; CTR on HCn-3 vs. CTR on HC, p = 0.007 (HC CTR n = 8; NEXLPL+/- n = 7, HCn-3 CTR n = 7; NEXLPL+/- n = 6). (C)(D) Morris Water Maze of NEXLPL+/- and CTR mice at 10–12 mo ages. Mice were fed with HC or omega-3 fatty acid enriched HC diet for 7–9 mo from 3 mo of age. Day4-8: hidden platform, learning phase. One way repetitive measure ANOVA showed diet difference only in HET on HCn-3 vs. HC, p = 0.003 (HC CTR n = 4; NEXLPL+/- n = 4 HCn-3 CTR n = 4; NEXLPL+/- n = 4). (E) Total saturated fatty acids (SFA) and total unsaturated fatty acids (UFA) in Chow and HC diets (Chow n = 2; HC n = 2, *, p<0.05).

With PUFA deficiency detected before the onset of neurobehavioral abnormalities in NEXLPL+/- mice, we fed PUFA-enriched diet (HCn-3 diet) to 18–20 mo NEXLPL+/- mice for 3 mo to test whether the learning and memory deficit in older NEXLPL+/- mice can be improved. Control HC diet were fed to a separate group of NEXLPL+/- mice, and both diets were fed to littermate CTR mice at the same age for similar period of time. At 21–23 mo of age after 3 mo of PUFA-enriched diet feeding, both NEXLPL+/- mice (Fig 6A, p = 0.07) and littermate CTR mice (Fig 6B, p = 0.007) appear to have improved learning in Morris Water Maze test. Interestingly, when younger NEXLPL+/- mice (3–5 mo) were fed with PUFA-enriched diet for a period of 7 months, 10–12 mo old NEXLPL+/- mice also showed improvement in the learning (Fig 6C, p = 0.003), while the CTR mice did not (Fig 6D).

The noticeable differences in their responses to PUFA enrichment diet for CTR mice at younger age vs. older age can be mostly attributed to the much poorer performance of CTR mice at older age (Fig 6B), where 21–23 mo CTR mice on HC control diet performed much poorer compared to those on chow (dotted darker line in Fig 6B representing the same data on chow diet as shown in Fig 2B, but re-plot here for direct comparison to solid darker line in Fig 6B for HC diet, p<0.001). Thus, after feeding HC diet for 3 mo, CTR mice now performed as poor as the NEXLPL+/- on HC control diet (Fig 6A). NEXLPL+/- on HC diet also performed poorer compared to those on chow (again, dotted darker line in Fig 6A vs. solid darker line in Fig 6A, p<0.001). But the effect of HC diet on NEXLPL+/- mice is less than CTR mice. At a much younger age (10–12 mo), CTR mice on HC diet performed better than NEXLPL+/- mice on HC diet (Fig 6D vs. Fig 6C darker lines). Furthermore, these data also implied that while NEXLPL+/- mice on chow diet at 13 mo of age did not show learning and memory deficit in previous Morris Water Maze test (Fig 2C), when these mice were fed HC diet at a much younger age (3–5 mo) for 7 mo, NEXLPL+/- mice did start to show learning and memory deficit at an earlier age than those fed on chow diet (Fig 6C).

Taken together, these data suggested that there might be similar underlying mechanism for the learning and memory deficit development in older WT control mice fed on HC diet and younger NEXLPL+/- mice fed on HC diet. To explore this further, we did lipidomic analysis of chow diet vs. HC diet. Compared to chow which has 18% total fat content, the reduction of total fat content to 10% in HC diet was not proportionally distributed among all FFA species (Fig 6E). Two essential fatty acids C18:2 (linoleic) and C18:3 (α-linolenic) are disproportionally deficient in HC diet vs. chow diet. When combining FFA species into saturated fatty acids (SFA), monounsaturated fatty acids (MUFA), and polyunsaturated fatty acids (PUFA) (Fig 6F), one can easily see that while the total SFA content difference in chow vs. HC diet generally reflected the total fat content difference in these two diets, the relative amount of PUFA is significantly deficient in HC diet.

In summary, several lines of evidences including: 1) a PUFA-deficient HC diet induced learning and memory deficit even in CTR control mice at an older age; 2) a PUFA-deficient HC diet accelerated learning and memory deficit development in NEXLPL+/- mice, and 3) a PUFA-enriched HCn-3 diet can improve learning and memory function for NEXLPL+/- at younger age and for both NEXLPL+/- and CTR controls at older age; all support the hypothesis that PUFA deficiency underlies the development of learning and memory deficit in NEXLPL+/- mice.

## Discussion

We investigated the behavioral phenotype of heterozygous neuron-specific LPL deficient mice (NEXLPL+/-) for two main reasons. First, NEXLPL+/- develop obesity with a similar but delayed time course compared to their homozygous counterparts. NEXLPL+/- also develop marked deficiency in hypothalamic PUFA levels preceding the development of obesity. The late life obesity development seen in NEXLPL+/- mice is reminiscent of age-related increase in obesity prevalence observed in humans. Second, the metabolic phenotype of NEXLPL+/- mice bears a greater clinical relevance in that homozygous LPL deficiency is highly uncommon in humans (1 in 1,000,000) and is characterized by a series of metabolic abnormalities including chylomicronemia, variable cognitive impairment, abdominal pain and often pancreatitis [30]. Heterozygous LPL deficiency is more common as it is estimated to be present in 3–7% of the general population [31–34], and is known to be associated with a greater risk of dyslipidemia, metabolic syndrome and atherosclerosis [35, 36]. Cognitive function has not been carefully evaluated in this population. Thus, NEXLPL+/- mice can serve as a preclinical model to study cognitive deficits and other behavioral alterations that might also be present in LPL heterozygous deficient human.

We have previously reported that NEXLPL+/- mice develop obesity at the age of 12 mo [13]; however, from our current study, NEXLPL+/- mice are not very different from CTR mice metabolically at 21–24 mo most likely due to the random spontaneous obesity development associated with aging in control mice. Interestingly, despite the trend to a higher weight and body fat percentage, NEXLPL+/- mice display a trend to having lower levels of circulating pro-inflammatory cytokines and pro- inflammatory gene expression in the hippocampus. These findings raise some question as to how the metabolic phenotype of NEXLPL+/- and CTR mice become similar as mice age. However in the context and the focus of characterizing behavioral abnormality in the current study, our data support the hypothesis that obesity is not the major cause of the cognitive or other behavioral abnormalities observed in NEXLPL+/- mice.

The behavioral tests we chose are well established models designed to target multiple aspects of the behavioral phenotypes that relate to different regions of the brain. Using these tests, NEXLPL+/- mice demonstrated deficits in learning and memory (Morris Water Maze and Novel Object Recognition) and increased anxiety (EPM). These tests implicate altered function in multiple brain regions including but not limited to the hippocampus, cortex, and amygdala. Because LPL reduction in the hippocampus is the most pronounced among all brain regions [13], and hippocampal function impairment has been implicated in at least two of the tests utilized in our study, it is reasonable to speculate that a deficit in hippocampal function due to LPL deficiency might underlie the behavioral abnormalities observed in NEXLPL+/- mice.

Although lipoprotein metabolism in mice is considerably different from that in humans in that the most abundant lipoprotein in mice is HDL, while in human, larger lipoproteins such as VLDL and LDL are more abundant, recent evidence from genetically-modified mice has clearly provided supports that alterations in brain lipoprotein metabolism can influence behavior. For example, the ApoE4 allele has been strongly associated with Alzheimer’s disease (review see [6]). Moreover, novel functions of lipoprotein receptors in CNS have emerged recently (recently reviewed in detailed by [9]). A study on mice with a neuron-specific knockout of the low-density lipoprotein receptor-related protein 1 (LRP1) revealed a neurodegenerative phenotype that was related to dendritic spine degeneration, synapse loss, and neuroinflammation [37]. LDLr knockout mice show increased locomotor activity, a decrease of learning and memory ability [38, 39], as well as impaired procedural memory [40]. Both VLDLr knockout and ApoEr2 knockout mice display contextual fear conditioning deficits and defects in long term potentiation (LTP) [41]. Also, a whole body LPL knockout mouse following somatic gene rescue demonstrated impaired learning and memory with a decreased number of presynaptic vesicles due to impaired recycling [11]. In addition, Nishitsuji K. et al. [12] reported that LPL is an Aβ binding protein that could facilitate Aβ clearing in astrocytes *in vitro*.

The data from neuron-specific LPL deficient mice support the importance of brain lipid metabolism in cognitive function and other neurobehavioral functions. Moreover, the primary function of LPL in peripheral tissues is to regulate TG-rich lipoprotein metabolism and lipoprotein-dependent fatty acid uptake and storage/oxidation [42]. The fact that the Aβ level in the brain of NEXLPL+/- mice is normal does not support an Aβ dependent pathway. Inflammation has also been implicated as a potential important contributor in neurodegenerative diseases, in which dopamine neuron loss and plaque formation are two major potential mechanisms [43, 44]. But with pro-inflammatory gene expression in the hippocampus and the circulating pro-inflammatory cytokines trending lower rather than higher in NEXLPL+/- mice, inflammation does not appear to be a significant contributor to the behavioral abnormalities in our model.

On the other hand, a synaptic plasticity deficit in the hippocampus indicated by reductions in both GluA1 total protein and Ser 845 phosphorylation in NEXLPL+/- could likely underlie the mechanisms of the behavioral abnormalities that we observe. Also, there is evidence that TNFα secreted by glia increases the expression of GluA1 AMPA receptors in both cultured hippocampal neurons and slices [45–47]. Of interest, we observed decreasing trends in the levels of TNFα in plasma and TNFα mRNA in hippocampus, which indicates that TNFα might play a role in LPL-regulated synaptic activity. An important question that remains to be answered pertains to the mechanisms through which LPL deficiency leads to lower levels of GluA1 protein and phosphorylation in neurons, and whether altered TNFα signaling is also involved in mediating these changes. We have provided evidence that a deficiency in omega-3 and omega-6 PUFA develops in the hippocampus before the appearance of behavioral abnormalities. It is thus reasonable to propose that the PUFA deficiency in the hippocampus underpins the impairment of hippocampal function in neuron-specific LPL deficient mice through the modification of GluA1 protein phosphorylation.

Indeed, PUFA deficiency has been associated with profound deficits in cognitive and other behavioral abnormalities in both rodents and humans. The CNS is highly enriched in long chain PUFAs of the omega-6 and omega-3 series, and most of these PUFAs are structural components of neuronal membranes, and serve as the sources of lipid-derived messengers. Dietary supplementation of omega-3 fatty acids has been reported to have beneficial effects in several types of diseases including heart disease [48] and visual loss [49]. In the CNS, increased DHA consumption has been shown to protect against dendritic pathology and loss in an AD mouse model [50], facilitate hippocampal development [51], and promote neurite growth [52]. More importantly, it has been reported that dietary supplementation with omega-3 PUFA can reverse the age-related impairment of LTP and glutamate transmitter release [53].

More relevant to the findings in this study, the potential effect of omega-3 PUFA on modifications of receptors that mediate neurotransmission has been reported in two separate cases previously. In one case, dietary enrichment with omega-3 PUFA such as eicosapentaenoic acid (EPA) and docosahexaenoic acid (DHA) reversed the age-related decreases in the glutamate receptor subunits GluA2 and GluN2B in rat forebrain [54]. In a cultured CA1 hippocampal slice experiment, AMPA receptor-mediated cell death was reduced by DHA but not by EPA [55], implying that dietary DHA uptake might be beneficial in preventing neurodegenerative diseases.

In the case of NEXLPL+/- mice, we have shown preliminary evidence that NEXLPL+/- mice fed on a relative PUFA-deficient HC diet (compared to chow) at young age (3-5mo) for a fairly extensive period (7 mo) display learning and memory deficit at 10–12 mo while those fed on chow display similar learning and memory deficits compared to controls, implying PUFA-deficient diet accelerates the learning memory deficit development in NEXLPL+/- mice. On the other hand, feeding of a PUFA-enriched diet to these NEXLPL+/- mice prevented the development of learning and memory deficit at 10–12 mo. Furthermore, feeding PUFA-deficient HC diet to much older control mice (18–20 mo) for only 3 mo significantly induced cognitive deficit in these mice, and feeding of PUFA-enriched diet to both older NEXLPL+/- and control mice improved the cognition at older age. It seems that mice that are deficient in PUFA earlier (as in younger NEXLPL+/- mice) will develop cognitive deficit earlier, but also respond to PUFA diet enrichment earlier. And at an older age (21–23 mo), all mice can benefit from PUFA-enrichment in the diet as age-dependent cognitive function decline is a general trend even in control mice.

In summary, we found that NEXLPL+/- mice develop neurobehavioral abnormalities which are similar to those described in the LPL-rescued whole body LPL deficient mouse [11], however the reduced AMPA receptor GluA1 protein levels and phosphorylation provides an additional mechanism. NEXLPL mice are a unique and novel model to study the potential importance of CNS lipoprotein metabolism in regulating behavioral functions without the interference of peripheral metabolic abnormalities [9]. The behavioral abnormalities we characterized in this study are age-dependent, involve multiple brain regions, proceeded by the PUFA-deficiency in hippocampus, and are potentially related to the loss of the AMPA receptor and its phosphorylation, eventually leading to reduced synaptic plasticity. Moreover, the reduced level of synaptophysin indicates that presynaptic function may also be impaired at hippocampal synapses in NEXLPL+/- mice. How overall synaptic function is altered and how the loss of AMPA receptor GluA1 expression and phosphorylation relates to LPL deficiency and/or PUFA deficiency will require further more detailed mechanistic studies. However, the promising result from NEXLPL+/- mice fed with PUFA-enriched diet suggests that dietary intervention can potentially be used as a prophylactic and therapeutic strategy for lipid-related neurodegenerative diseases such as Alzheimer’s Diseases.
